# Disparities in preventive procedures: comparisons of self-report and Medicare claims data

**DOI:** 10.1186/1472-6963-6-122

**Published:** 2006-09-29

**Authors:** Kevin Fiscella, Kathleen Holt, Sean Meldrum, Peter Franks

**Affiliations:** 1 Department of Family Medicine, University of Rochester School of Medicine and Dentistry, Rochester, New York, USA; 2 Center for Health Serv Res in Primary Care, Department of Family and Community Medicine, University of California School of Medicine, Davis, Sacramento, California, USA

## Abstract

**Background:**

Racial/ethnic disparities are assessed using either self-report or claims data. We compared these two data sources and examined contributors to discrepancies in estimates of disparities.

**Methods:**

We analyzed self-report and matching claims data from Medicare Beneficiaries 65 and older who participated in the Medicare Current Beneficiary Survey, 1999–2002. Six preventive procedures were included: PSA testing, influenza vaccination, Pap smear testing, cholesterol testing, mammography, and colorectal cancer testing. We examined predictors of self-reports in the absence of claims and claims in the absence of self-reports.

**Results:**

With the exception of PSA testing, racial/ethnic disparities in preventive procedures are generally larger when using Medicare claims than when using patients' self-report. Analyses adjusting for age, gender, income, educational level, health status, proxy response and supplemental insurance showed that minorities were more likely to self-report preventive procedures in the absence of claims. Adjusted odds ratios ranged from 1.07 (95% CI: 0.88 – 1.30) for PSA testing to 1.83 (95% CI: 1.46 – 2.30) for Pap smear testing. Rates of claims in the absence of self-report were low. Minorities were more likely to have PSA test claims in the absence of self-reports (1.55 95% CI: 1.17 – 2.06), but were less likely to have influenza vaccination claims in the absence of self-reports (0.69 95% CI: 0.51 – 0.93).

**Conclusion:**

These findings are consistent with either racial/ethnic reporting biases in receipt of preventive procedures or less efficient Medicare billing among providers with large minority practices.

## Background

Racial and ethnic disparities in preventive procedures have been widely documented [1-9]. These findings are based primarily on either patient self-report or the use of claims data. Each has limitations. Self-report is associated with significant overestimation of rates of preventive procedures in most [10-17], but not all studies [18,19]. In contrast, claims data may underestimate procedures [20]. The size of disparities in mammography differs depending on survey question wording [21,22], but more importantly, on whether self-report or claims data are used [23]. Such discrepancies could represent either greater over-reporting by minorities of preventive procedures relative to majorities or less efficient Medicare billing procedures by providers servicing minority patients.

Determining whether different data sources yield disparate estimates of racial and ethnic disparities in preventive procedures is relevant to national monitoring of these disparities. A finding of similar disparities regardless of data source reinforces use of self-report data by the National Healthcare Disparities Report or for tracking progress towards Health People 2010 objectives. Discrepant findings suggest the need for further research to determine which is the more reliable data source for assessing disparities. Assessment of potential discrepancies is also critical to understanding disparities in health. Self-report data from the National Health Interview show small racial disparities in mammography [24], but analysis of Medicare claims show significantly larger racial disparities [23]. Significantly, racial disparities in mammography based on Medicare claims have been linked to racial differences in stage at diagnosis [25].

Thus, the primary aim of this study is to determine whether estimates of racial and racial disparities in receipt of six different types of largely preventive procedures differ between self-report and Medicare claims data. To do so, we determine whether minority status, defined as African American or Hispanic (compared to non-Hispanic White) is associated with self-report of procedures in the absence of a claim and vice versa. We also examine whether any such associations are accounted for by other patient characteristics.

Throughout this paper we use the term preventive procedures rather than screening to indicate that while the majority of procedures are likely performed for screening purposes, some are performed for diagnostic or other reasons. Most published studies analyzing self-report data do not distinguish the reason for the procedure, and typically assume the procedure is for screening. Most national self-report surveys (including the Medicare Current Beneficiary Survey used here, the Medical Expenditure Panel Survey, and the Behavioral Risk Factor Surveillance System) do not have a question on the reason for testing. While the National Health Interview Survey currently does include a question on the reason for testing, the information is not used in the published procedure rates used to track the Healthy People 2010 goals [24], or in publications derived from the National Health Interview Survey [3,26,27]. Our analyses examine discrepancies between self-report and claims data, so we use an inclusive definition of preventive procedures to enable comparison to other self-report literature.

## Methods

### Data sources

The study was approved by the University of Rochester Human Subjects Review Board. Data used were the Centers for Medicare & Medicaid Services' (CMS) Medicare Current Beneficiary Survey (MCBS). The MCBS includes an annual survey (for a maximum of four years per participant) to a rotating panel of Medicare beneficiaries. Participants are asked to recall tests and procedures they underwent during the proceeding year. After the first year, participants are given diaries to augment their memories; however, no information is available as to how widely these diaries are used by participants and self-reports are based on verbal response rather than examination of diaries. Selected subpopulations are over-sampled and appropriate longitudinal and cross-sectional weights are provided to allow for estimates for the entire Medicare population.

Medicare claims data (diagnoses, and diagnostic and procedural medical services) are also available for each year respondents participate and can be linked to the survey data. This linkage at the patient level allows for direct comparison between participants' self-report of services received and those documented by the existence of Medicare claims. Further details about the survey are available at the CMS website[28]

To yield sufficient power, survey, physician/supplier, and outpatient data were aggregated across four years (1998–2002) where possible. However, questions about cholesterol testing were asked only in 2001 and 2002, and questions about colorectal cancer testing only in 2000. Subjects' first year of participation in the survey was eliminated because a full year of corresponding claims in the preceding year is not available for first year participants in MCBS. Thus, comparison between self-report and existence of complete claims is available only for those completing at least 2 years of the survey. In analyses not reported, results consistent with those below were obtained using the nine months of claims data prior to the first survey year. The total number of observations available from 2 to 4 years of surveys was 88,509.

### Exclusion criteria

Respondents were excluded from our sample if they participated in facility interviews (i.e., resided in long-term care facilities, n = 6,462), were less than 65 years of age (i.e., were Medicare recipients due to having a qualifying disability, n = 12,852), reported race/ethnicity *other than *Hispanic, non-Hispanic African American, or non-Hispanic White, i.e. majority (n = 3,169 dropped due to small sample size of other race/ethnicities), were enrolled in a Medicare HMO (n = 15,262 dropped because claims were not available from medical encounters), or were not eligible for Medicare B (or Medicare A and B) coverage (n = 1,118) dropped due to incomplete claims. The resulting analytic sample contained 42,949 majority, 4,168 African American, and 2,528 Hispanic observations.

### Measures

#### Race/Ethnicity

Minority status was defined as self-report of African American/Black race or Hispanic ethnicity: based on the responses to two questions: "(Are you/Is SP) of Hispanic or Latino origin?" [Yes or No] ; and "Looking at this card, what is (your/SP's) race?" [American Indian or Alaska Native; Asian; Black or African American; Native Hawaiian or other Pacific Islander; White; Another Race (Specify)]. Participants in our analyses were classified as a minority if they responded either "Yes" to the first question or "Black or African American" to the second question. This aggregate measure was used in the primary analyses to increase power; secondary analyses were conducted separately for African Americans and Hispanics.

#### Receipt of preventive procedures

The questions and corresponding CPT, HCPC, and Berenson-Eggers Type of Service (BETOS) codes used to identify persons receiving preventive procedures are shown in Table 1. Because of challenges in distinguishing screening procedures from diagnostic procedures [20], both screening and diagnostic codes were included. This was necessary to ensure appropriate comparison between self-report and claims data, as the self-report questions did not distinguish tests done for screening purposes from those done diagnostically.

To assess self-reported colorectal cancer testing, the combination of two measures (either submitting a kit for fecal occult blood testing or undergoing a sigmoidoscopy or colonoscopy in the past year) was used. Sigmoidoscopy was combined with colonoscopy because the self-report question did not distinguish them. Secondary analyses were also conducted separately for sigmoidoscopy/colonoscopy and fecal occult blood testing.

#### Covariates

Analyses adjusted for the following MCBS respondent characteristics: age (categorized as: 65–69, 70–74, 75–79, 80–84, with 85 and older as the reference group), education (less than high school graduation vs. at least high school graduation), annual income (less than $25,000 vs. $25,000 or more), metropolitan residence (vs. not), whether the respondent lived alone (vs. not), availability of supplemental insurance (private insurance, Medicaid supplemental, vs. none), proxy response to the survey (vs. self-response), functional status – Activities of Daily Living scale (a 3-point impairment scale) [29], and respondents' estimate of their general health compared to others of their age (5-point scale). This type of comparative self-rating of health status has been shown to predict mortality [30,31].

### Statistical analyses

To accommodate the complex survey design of the MCBS, including the multiple years of enrollment in the survey, SAS SURVEY procedures were used (SAS Institute, Cary, NC, Version 9.1, 2002–2003,). Survey weights were used to adjust for over-sampling and non-response to yield population parameter estimates. Data were analyzed with logistic regression to assess the adjusted relationship between minority status and self-report in the absence of a claim, as well as for a claim in the absence of a self-report.

## Results

Table 2 summarizes the characteristics of respondents asked about each preventive service. The sample sizes for each differ depending on how many years the survey question was asked and whether the procedure was relevant to men, women, or both.

**Figure 1:**
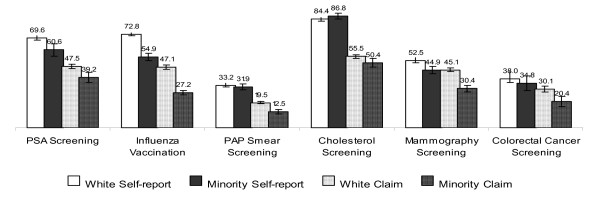
Unadjusted percent* of respondents, by minority status, with a self-report or claim for a preventive procedure. * Horizontal lines at the top each bar refer to 95% confidence intervals surrounding the estimate.

The comparisons of self-reported and claims-based preventive procedures, by minority status, are shown in Figure 1. Rates of preventive procedures based on self-report are significantly higher than those based on claims, for both minorities and majorities. Absolute differences by minority status (majority vs. minority) for self-report ranged from -2.4% for cholesterol testing to 17.9 % for influenza vaccination. In contrast, differences based on claims data ranged from 5.1% for cholesterol testing to 19.9 % for influenza vaccination.

Overall agreement between self-report and claims was generally lower for minorities. The kappa statistic measures the level of agreement two values with values ranging from "0" for no agreement to "1.0" for perfect agreement. A kappa between self-report and claims data for minorities ranged from 0.19 (for colorectal cancer testing) to 0.58 (for mammography). For majorities, these statistics ranged from 0.37 (for colorectal cancer testing) to 0.70 (for mammography). The different ranges of kappa between majorities and minorities are largely due to higher rates of self-reported preventive procedures in the absence of a claim for minorities.

Logistic regression analyses adjusting for age, gender, income, educational level, health status, proxy response and supplemental insurance resulted in relatively little change in the odds ratios by minority status for reporting a procedure in the absence of a claim or vice versa (Table 3). Minorities were more likely than majorities to report receipt of a preventive procedure in the absence of a claim. Adjusted odds ratios ranged from 1.07 (95% CI: 0.88 – 1.30) for PSA to 1.83 (95% CI: 1.44 – 2.30) for Pap smear testing. Secondary analyses conducted separately for African Americans and Hispanics yielded similar findings for self-report in the absence of a claim. Analyses conducted separately for the different modalities of colorectal testing also gave similar results.

Rates of a claim in the presence of self-report of *non*-receipt were appreciably lower than self-report in the absence of a claim. Again, the crude and adjusted odds ratios were similar (Table 3). Minorities were more likely than Majorities to have a PSA test claim in the absence of a self-report (1.55 95% CI: 1.17 – 2.06), but were less likely to have an influenza vaccination claim in the absence of a self-report (0.69 95% CI: 0.51 – 0.93). The remaining odds ratios did not reach statistical significance.

To address the differences in report of true preventive services versus diagnostic services, analyses for PSA testing, mammography, and colorectal cancer testing were recomputed using only true preventive codes [20]. Those models produced results very similar to models described above: similar minority effects were apparent for mammography and colorectal cancer testing; no minority effects were observed for PSA testing.

## Discussion

Previous research has shown larger disparities in mammography between African Americans and majorities when receipt of mammography is based on claims data rather than on self-report [23,32]. The current study extends these findings to other preventive procedures. With the exception of PSA testing, greater discrepancies were observed between self-reported receipt of preventive care and documentation by Medicare claims among minorities. These effects persisted after controlling for a range of patient characteristics and were observed across different types of preventive procedures. These discrepancies resulted in differing estimates of racial/ethnic disparities in preventive care with generally larger disparities observed using claims than self-report data.

Unfortunately, there is no clear gold standard in this study; we cannot determine whether self-report or a Medicare claim is a more accurate reflection of procedures received. It is not clear whether higher rates of self-report in the absence of a claim represent a greater tendency among minorities to "over-report" across many procedures or whether these findings represent suboptimal Medicare billing by providers who serve minorities.

Each of these explanations has some plausibility. Based on medical record documentation, self-report tends to overestimate actual receipt of preventive procedures partly through underestimation of the time interval since the previous test [12,14,15,17,33,34]. Some studies suggest this reporting bias may be greater among minorities than among majorities [13,14,35,36]. Moreover, reports of preventive care by minorities seem to be more sensitive than non-minority reports to survey question wording [21,22]. Potential explanations for these examples of over-reporting include cultural differences in perception of time elapsed, effects of cross racial/ethnic interviewer-respondent effects [37-39], and social desirability [40-43].

While self-reports overestimate receipt of preventive procedures, administrative billing data may underestimate actual use [16]. Minorities are more likely to been seen by safety net providers such as hospital clinics and community health centers [44]. These providers often receive substantial revenue through Medicaid or Medicare prospective payment system [45,46]. As a result, there may be less financial incentive among these providers to optimize patient Medicare encounter billing since much of their patient revenue is not as strongly dependent on billing coding for specific visits [47]. Furthermore, greater use of paper submission of claims or slower adoption of Medicare prevention codes [20] could result in higher claim rejection rates by Medicare. This, in turn, could affect data sources that rely on the presence of claims to document receipt of procedures.

There are several ways these different explanations could be distinguished in future research. Rates of self-reported receipt of preventive procedures and Medicare claims data by race or ethnicity could be compared based on chart validation or through other sources of validation. For example, Medicare claims for mammography were recently compared to a mammography registry showing claims data to be moderately sensitive [48]. In addition, studies are needed to compare Medicare billing patterns including payment denial according to the racial composition of the physician's practice. For example, a finding of higher preventive procedure claim denial rates for those physicians' practices having large numbers of minorities would suggest that the use of claims data may be biased for those practitioners.

Distinguishing among these competing explanations has important policy implications. If, on one hand, minorities are more likely to "over-report" receipt of preventive procedures, estimates of racial/ethnic disparities in those procedures based on survey self-report will underestimate the actual magnitude of disparities. This could result in the premature conclusion that disparities in preventive care have been eliminated. For example, self-report survey data have shown that African American women have caught up with majority women in most areas of preventive procedures and actually exceed them in others [8,49]. Differences in survey wording appear to reduce disparities by reducing reporting bias associated with minority status [21]. Increasing use of electronic health records may eventually minimize reliance on billing and survey data to assess disparities in preventive care.

If, on the other hand, if these discrepancies are primarily driven by inconsistencies in billing, this suggests caution in the use of claims data, not only as a proxy for procedures performed, but for comparisons among providers who serve a greater proportion of minority patients. That is, differential billing efficiency may bias comparisons among different types of providers [50]. Such bias could penalize minority care providers, as many current pay-for-performance systems rely on claims data to assess care and reward performance. Further, failure to successfully bill for procedures could result in decreased payments, and thus compromise resources available to provide quality care.

The limitations of our analyses should be noted. First, the sample was confined to community dwelling, Medicare beneficiaries aged 65 and older, and those not enrolled in managed care. The extent to which these findings generalize to other groups cannot be assessed. Second, colorectal cancer testing survey questions were only asked in one year. As a result, the small sample size limited the power of these analyses. Third, although the findings summarized in Figure 1 suggest a general pattern of larger disparities based on claims compared to self report data, the discrepancies are relatively modest and beyond the power of the study to demonstrate statistically significant difference in differences. Fourth, we used a broad definition of preventive procedures because these data do not allow us to clearly distinguish between screening or diagnostic tests. However, comparable effects to those presented above were found when analyses were repeated using preventive codes only. Fifth, and finally, our selection of procedures might be differentially affected by the systematic lack of claims. For some procedures and selected years, Medicare did not reimburse for screening, e.g., Pap smear testing is reimbursed biennially and reimbursement for PSA screening and colonoscopy did not begin in 2001. As a result, physicians may have billed for screening services using diagnostic codes (which is why we included both codes). However, limitation of analyses to 2001 and/or exclusion of colonoscopy did not appreciably alter the findings.

Embedded in the problem of distinguishing screening and diagnostic testing is a critical issue for monitoring disparities in screening. Some claims-based evidence suggests that disparities for diagnostic testing are narrower than those for screening [5]. Such evidence is consistent with the observation that minority patients present later in the course of cancer [51] (and thus obtain "diagnostic" rather than "screening" tests). Thus, self-report studies that, in general, do not distinguish screening and diagnostic testing may underestimate disparities in screening.

## Conclusion

This study shows that estimates of racial/ethnic disparities, across a variety of preventive care procedures, vary depending on whether self-report or claims are used to assess them. Whether these differences reflect biases in participant report or in billing claims is unclear. These competing explanations have profoundly different policy implications, and thus warrant careful study. Future monitoring of disparities in screening will require more careful distinction of screening from diagnostic uses of preventive procedures.

## Competing interests

The author(s) declare that they have no competing interests.

## Authors' contributions

KF conceived the project, obtained grant funding, directed the analyses, participated in the analyses and interpretation of the results, and took the lead in the manuscript writing. KH conducted the analyses, participated in the interpretation of the results and manuscript writing. SM participated in the analyses and participated in writing. PF participated in the conception of the study, analyses, interpretation and writing. All authors read and approved the final manuscript.

## Pre-publication history

The pre-publication history for this paper can be accessed here:



## Figures and Tables

**Table 1:** Preventive procedure questions and CPT, HCPC, and BETOS codes

**Preventive Procedure**	**Survey Question**	**Codes**
PSA testing	Have you a blood test for the detection of prostate cancer, known as PSA, in the past year?	84153, 84154, G0103
Influenza vaccination	Did you have a flu shot for last winter?	90732, 90724, 90659, 90658, 90669, G0008, or a BETOS code of O1G
Pap smear testing	Have you had a PAP smear in the past year?	G0101, G0124, G0141, Q0091, P3001, G0123, G0143, G0144, G0145, G0147, G0148, 88142, 88143, 88147, 88148, 88150, 88151, 88152, 88153, 88154, 88156, 88157, 88164, 88165, 88166, 88167, 88174, 88175, P3000
Cholesterol testing	When was the last time you had your blood cholesterol taken [Yes in the past year]?	82465, 83718, 83721, 83719, 80061.
Mammography	Have you had a mammogram in the past year?	76090, 76091, 76092, or a BETOS code of I1C
Colorectal cancer testing	When was your most recent test done? [Yes in the past year to EITHER colonoscopy test OR blood stool test]	82270, 82272, 82274, 82270, G0328, G0107, or a BETOS code of P8C or P8D

**Table 2:** Percentages of sample, by socio-demographic and survey characteristics

	PSA Testing	Influenza Vaccination	Pap Smear Testing	Cholesterol Testing	Mammography	Colorectal Cancer Testing*
	N = 7,009	N = 18,315	N = 4,784	N = 10,421	N = 7,404	N = 1,474
Minority						
Hispanic, African American	10.1	9.9	13.3	13.2	11.8	8.8
Majority	89.9	90.1	86.7	86.8	88.2	91.2
Age						
65–69	16.4	14.2	19.7	15.5	17.9	16.7
70–74	35.2	30.6	34.6	31.1	33.8	35.1
75–79	24.9	25.6	25.3	25.0	25.6	26.7
80–84	15.4	17.1	13.4	16.6	15.0	14.3
85 +	8.2	12.5	7.1	11.8	7.8	7.2
Education						
<HS graduate	26.7	29.1	25.1	30.3	25.8	23.5
>=HS Graduate	73.3	70.9	74.9	69.7	74.2	76.5
Metropolitan Area						
No	27.2	29.5	25.2	27.1	26.5	26.6
Yes	72.8	70.5	74.8	72.9	73.5	73.4
Income						
<=25,000 annual	43.4	56.6	59.0	56.9	59.9	50.3
>25,000 annual	56.6	43.4	41.0	43.1	40.1	49.7
Lives Alone						
No	82.9	67.5	60.1	67.2	59.1	69.8
Yes	17.1	32.5	39.9	32.8	40.9	30.2
Proxy Responded						
No	90.2	92.5	96.7	92.2	96.8	94.2
Yes	9.7	7.4	3.2	7.8	3.1	5.8
Supplemental insurance						
None	12.5	10.1	8.4	12.7	7.7	8.3
Medicaid	4.2	7.5	8.7	9.0	8.7	5.5
Private Insurance	83.3	82.3	82.8	78.3	83.5	86.2
MCBS Survey Years						
2	36.5	37.1	38.6	37.1	37.6	39.3
3	32.8	32.5	32.3	33.0	33.1	31.7
4	30.6	30.4	29.1	29.9	29.3	29
Activities of Daily Living^1^	1.32	1.54	1.43	1.54	1.48	1.41
Self-assessed health status^2^	2.67	2.73	2.63	2.75	2.65	2.70
Sex						
Male	100	42.1	0	41.8	0	43.8
Female	0	57.90	100	58.2	100	56.2

^1 ^On a 3-point scale, with higher scores indicating more impairment.

^2 ^On a 5-point scale, with higher scores indicating poorer health.

* Includes fecal occult blood testing, sigmoidoscopy, or colonoscopy.

**Table 3:** Effect of Minority Status on reported receipt of preventive health care procedures, in the absence of a corresponding claim, and a claim in the absence of a self-report.

**Odds Ratios for Minorities Compared to Majorities**^**a**^						
	PSA Testing	Influenza Vaccination	Pap Smear Testing	Cholesterol Testing	Mammo- graphy	Colorectal Cancer Testing
**Self-Report in the Absence of Claim**						

**Crude Odds Ratio**	1.38	1.90	2.09	1.36	2.09	1.92
**Sample Sizes**	8,756	23,776	6,111	10,780	9,329	1,484
**95% CI**	(1.20–1.59)	(1.74–2.08)	(1.78–2.46)	(1.21–1.53)	(1.82–2.39)	(1.32–2.79)
**Adjusted Odds Ratio**	1.07	1.64	1.83	1.20	1.75	1.68
**Sample Sizes**	7,009	18,315	4,784	10,421	7,404	1,474
**95% CI**	(0.88–1.30)	(1.45–1.85)	(1.46–2.30)	(1.02–1.42)	(1.40–2.18)	(1.23–2.29)

**Claim in the Absence of Self-report**						

**Crude Odds Ratio**	1.37	0.73	0.70	1.01	0.66	1.25
**Sample Sizes**	4,673	9,897	13,433	1,970	10,320	867
**95% CI**	(1.11–1.70)	(0.57–0.93)	(0.52–0.95)	(0.67–1.53)	(0.53–0.82)	(0.66–2.39)
**Adjusted Odds Ratio**	1.55	0.69	0.89	1.28	0.78	1.36
**Sample Sizes**	3,366	7,541	10,158	1,906	7,612	860
**95% CI**	(1.17–2.06)	(0.51–0.93)	(0.63–1.26)	(0.79–2.08)	(0.59–1.03)	(0.66–2.81)

^a^Odds ratio reporting receipt of procedure in the absence of claim or vice versa, adjusted for age, education level, health status, proxy response, metro-area residence, supplementary insurance, years participating in survey, and, where appropriate, sex.
